# Thyroxine binding to type III iodothyronine deiodinase

**DOI:** 10.1038/s41598-020-72243-9

**Published:** 2020-09-21

**Authors:** Craig A. Bayse, Eric S. Marsan, Jenna R. Garcia, Alexis T. Tran-Thompson

**Affiliations:** Department of Chemistry and Biochemistry, Old Dominion University, Norfolk, VA 23529 USA

**Keywords:** Thioredoxins, Computational chemistry

## Abstract

Iodothyronine deiodinases (Dios) are important selenoproteins that control the concentration of the active thyroid hormone (TH) triiodothyronine through regioselective deiodination. The X-ray structure of a truncated monomer of Type III Dio (Dio3), which deiodinates TH inner rings through a selenocysteine (Sec) residue, revealed a thioredoxin-fold catalytic domain supplemented with an unstructured Ω-loop. Loop dynamics are driven by interactions of the conserved Trp207 with solvent in multi-microsecond molecular dynamics simulations of the Dio3 thioredoxin(Trx)-fold domain. Hydrogen bonding interactions of Glu200 with residues conserved across the Dio family anchor the loop’s N-terminus to the active site Ser-Cys-Thr-Sec sequence. A key long-lived loop conformation coincides with the opening of a cryptic pocket that accommodates thyroxine (T_4_) through an I⋯Se halogen bond to Sec170 and the amino acid group with a polar cleft. The Dio3-T_4_ complex is stabilized by an I⋯O halogen bond between an outer ring iodine and Asp211, consistent with Dio3 selectivity for inner ring deiodination. Non-conservation of residues, such as Asp211, in other Dio types in the flexible portion of the loop sequence suggests a mechanism for regioselectivity through Dio type-specific loop conformations. Cys168 is proposed to attack the selenenyl iodide intermediate to regenerate Dio3 based upon structural comparison with related Trx-fold proteins.

## Introduction

**Figure 1:**
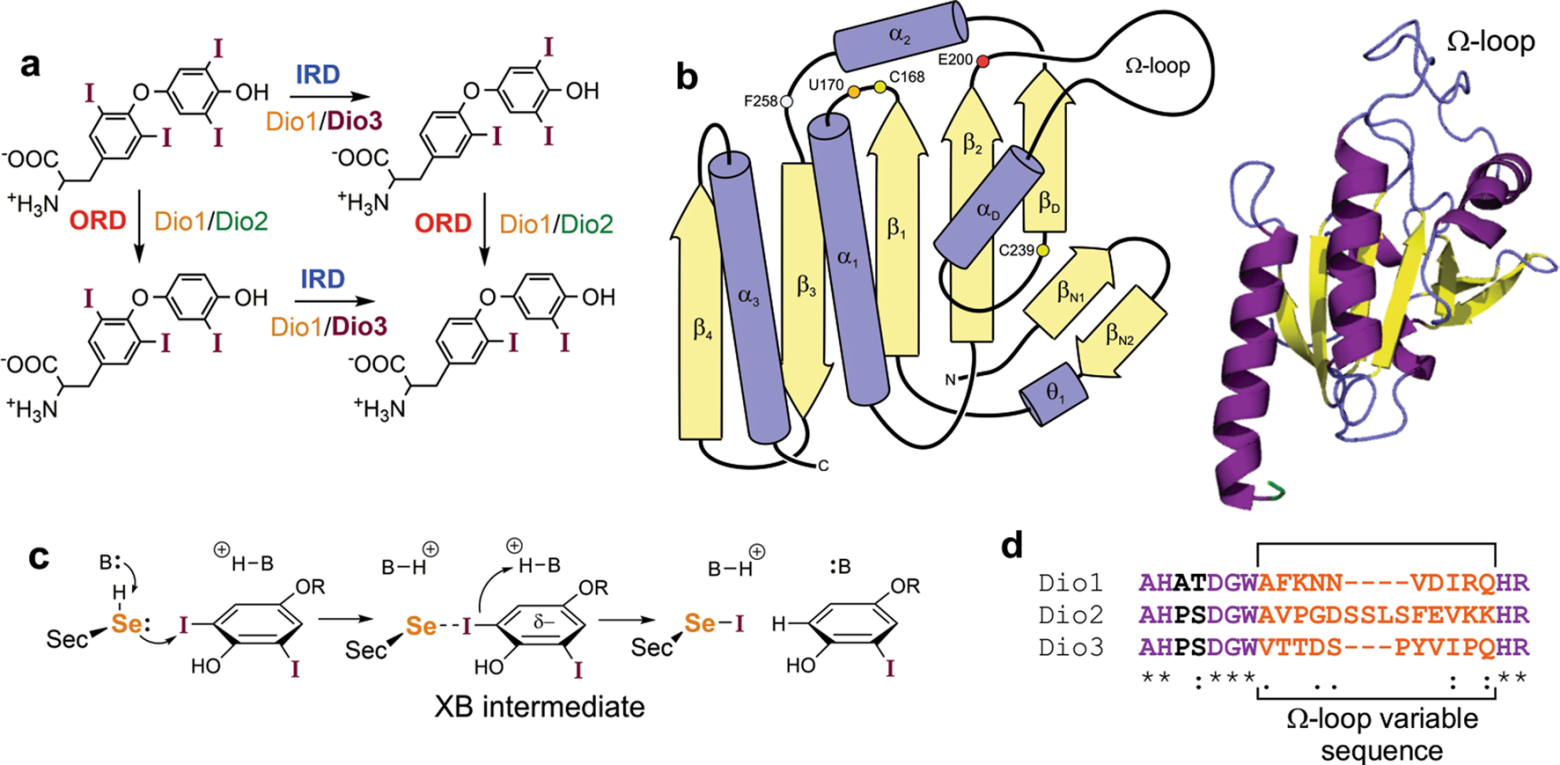
Structure and mechanism of Dio. (**a**) Regioselectivity of deiodination by Dio (**b**) Schematic diagram and X-ray structure (PDB 4TR4) of the Trx-fold catalytic domain of *mus* Dio3. Cartoon image created using VMD 1.9.3. (**c**) Halogen bonding mechanism for deiodination. The bases (B) that facilitate proton transfer have not been determined (**d**) Sequence alignment of the Ω-loop in *mus* Dio1-3. Contrast the high similarity on the termini with the variability in sequence and length for the central portion.

Iodothyronine deiodinase (Dio) membrane selenoproteins regulate thyroid hormone (TH) activity through regioselective deiodination (Fig. 1a)^1–10^. The thyroid gland primarily produces the prohormone thyroxine (3,3′,5,5′-tetraiodothyronine, T_4_), which is distributed to peripheral tissues for outer ring deiodination (ORD) to the active TH 3,3′,5-triiodothyronine (T_3_) by Type II (Dio2)^3,11^. Type III (Dio3) maintains TH homeostasis by removing iodine from the inner ring (IRD) of T_4_ or T_3_ to produce inactive reverse-T_3_ (3,3′,5′-triiodothyronine, rT_3_) or 3,3′-diiodothyronine (3,3′-T_2_), respectively^5,6,12^. Type I (Dio1) deiodinates either ring, but prefers ORD^1^. Structural studies of Dio are challenging due to the difficulty of isolating membrane proteins^13^. By removing the N-terminal transmembrane region, Schweizer et al. solved the first structure of the catalytic domain of *mus* Dio3^14^. The thioredoxin(Trx)-fold motif of the monomer was shown to be supplemented with an N-terminal β_1_ extension related to peroxiredoxins (Prx) and a Dio-insertion region between β_2_ and α_2_ (Fig. 1b)^14,15^. The characteristic CXXC active site motif at the β_1_α_1_ turn of Trx-fold proteins is modified to Ser-Cys-Thr-Sec (SCTU), incorporating a highly nucleophilic selenocysteine (Sec, U)^16,17^.

The Dio deiodination mechanism involves an initial I⋯Se halogen bonded (XB) intermediate complex of Sec170 and the TH substrate (Fig. 1c)^18–22^. From this intermediate, the protein facilitates protonation of the carbanionic leaving group for an overall nucleophilic attack of selenolate on the C–I bond. The selenenyl iodide (-SeI) intermediate reacts with an internal resolving Cys to a selenosulfide and Dio is regenerated through reduction by dithiothreitol in vitro or an endogenous thiol cofactor^23^. Endocrine disrupting halogenated aromatic compounds such as polychlorinated biphenyls (PCBs) and polybrominated diphenyl ethers (PBDEs) could inhibit Dio by forming a X⋯Se complex that blocks TH access to Sec^24–26^. Although XB interactions differ in strength for inner and outer-ring iodines when calculated by density functional theory (DFT)^24,27^, the regioselectivity of deiodination (IRD vs ORD) must be controlled by interactions between the substrate and active site residues^24^. However, no direct structural information is available for a TH or inhibitor complex with Dio that would demonstrate how individual Dio types control selectivity.

The X-ray structure for the catalytic domain of the *mus* Dio3 monomer shows an unstructured β_2_α_D_-loop (loop-D) adjacent to the active site within the Dio-insertion site^14^. Extended loops of 19–23 amino acids are a common feature of Dio proteins. Ω-loops, first described by Fetrow (Lezsczynski) et al., are irregular secondary protein structural motifs^28–30^ that lack repeating backbone dihedral or hydrogen bonding patterns^28^. These features are commonly found on the exterior of proteins, suggesting a role in substrate binding, catalytic activity, or protein stability^28,29^. Loop-D of *mus* Dio3 (residues 201–217: AHPSDGWVTTDSPYVIP) contains residues (e.g., Pro, Asp, and Gly) and other structural features common to Ω-like loops, such as sequence length; lack of regular secondary structure; and a short Cα–Cα distance between the segment termini (3.7–10.0 Å)^28,30^. While the Trx-fold and the N-terminal AHxxDGW sequence of the β_2_α_D_ loop is conserved across the Dio family, the length and sequence of the Ω-loop varies significantly while maintaining high similarity within types (Fig. 1d) suggesting a potential contribution to Dio regioselectivity. Of this sequence, His202 (corresponding to His162 for Dio2 and His158 for Dio1 in *Mus musculus*) was proposed to bind the 4′-phenol of THs^14^, but the role of other conserved residues have not been established. The constriction point of the Ω-loop is a hydrogen bonding interaction between Ser204 NH and Ile216 CO (d(Cα–Cα) = 5.26 Å).

Multi-microsecond molecular dynamics (MD) simulations of the monomer of the Dio3 Trx-fold domain identify a key conformation the Ω-loop that coincides with the opening of a cryptic pocket to the active site Sec170. In simulations with T_4_ docked to this pocket and modelled with a strong inner-ring I⋯Se interaction, substrate binding is supplemented by a I⋯O interaction between an outer-ring iodine and the Asp211 loop residue, consistent with the regioselectivity of Dio3. Hydrogen bonding interactions and packing of nonpolar groups such as Trp207 into the interior of the loop contribute to the conformation dynamics of the loop. Extending this understanding of the role of the Ω-loop and XB interactions to Dio3-T_4_ binding to both the mechanism and selectivity could lead to development of targeted treatments for thyroid hormone-related disease.

## Results

### Conformation dynamics in Dio3^trunc^

**Figure 2:**
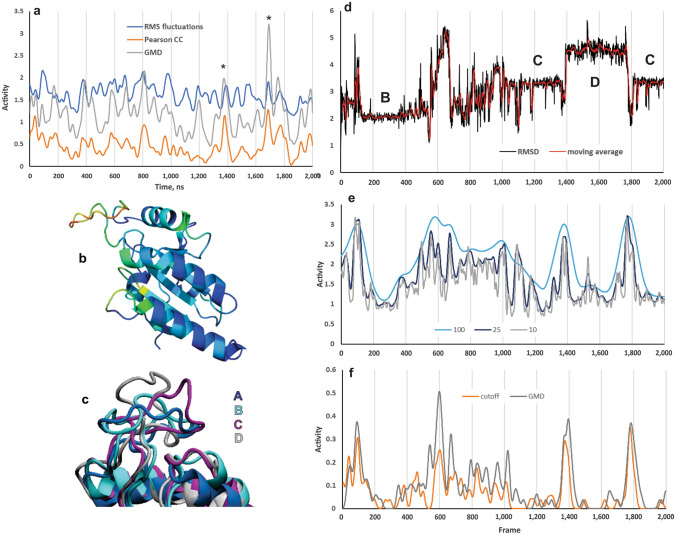
Statistical analysis of the Dio3^trunc^ trajectory. (**a**) Activity measures calculated using the RMS fluctuation (median filtering with a sliding window of 25 frames), cutoffs (6 and 8.5 Å) and GMD (order 2 and 3). (**b**) Superposition of the residue pairwise contact heat map on the X-ray structure of Dio3^trunc^. The fast motions of the Ω-loop (red) contrast with the slow dynamics (blue) of the Trx fold. Image created using TimeScapes version 1.5 and Pymol version 1.10.10. (**c**) Overlay of the X-ray structure **A** and key loop conformations **B**–**D** in MD simulations of Dio3^trunc^. Image created using VMD version 1.9.3. (**d**) Traditional RMSD of the Ω-loop calculated in terms of Cα for the loop-only culled trajectory. (**e**) Comparison of filtering on the RMS fluctuations using various sliding window lengths (100, 25, 10). (**f**) Event detection activity determined by cutoff- (6.0–8.5 Å crossing buffer) and GMD-based (order 2,3 crossing buffer) screening using the 25-frame screened trajectory.

MD simulations of the Trx-fold region of monomeric Dio3 were performed for 20 μs starting from the X-ray structure **A** (PDB: 4TR4)^14^ truncated to Gly132 (Dio3^trunc^). The Ω-loop conformation of **A** is found to be unstable because the nonpolar sidechains (i.e., Trp207, Val215, Pro217) on the exterior loop surface reorganize in search of a more favourable solution-phase conformation. The structure of the Trx-fold is generally constant during simulations with significant fluctuations found in the β_2_α_D_ Ω-loop which adopts three long-lived conformations (**B**–**D**, Fig. 2 and S1). These results are consistent with normal mode analysis^31^ of the X-ray structure which indicates that the motion of the active site SCTU sequence and the Ω-loop are slightly correlated (See Supporting Information).

Statistical analysis was used to map residues with fast motion relative to the protein's global dynamics by determining pairwise contact events and backbone hinge motions (Fig. 2)^32,33^. Median filtering of the root-mean-square (RMS) fluctuations reflects the random distribution of events within the bulk of the Trx fold of Dio3^trunc^ over the course of the trajectory (Fig. 2a). Distance cutoff and generalized masked Delaunay (GMD) buffer regions select for significant events, such as the reversible **C**–**D** transitions, while screening out trivial events (Fig. 2a)^32,33^. Most pairwise contact events involve the residues of the Ω-loop, especially the conserved Trp207 (Figs. 2b, S3). The magnitude of the activity within RMS fluctuation (RMSF) mapping is consistent with the raw Cα RMSF (Fig S1b). Other regions with significant contact event activity are also found at the flexible β_N1_β_N2_, θ_1_β_1_, and α_D_β_D_ turns.

Separate analysis of a culled trajectory of Ω-loop residues 201–210 (Fig. 2d–f) shows that the highest portion of events occur within the transition regions between conformations with a sharp increase at the **C**–**D** conformation changes. Hinge-bending motions are prevalent for the pivot angles of residues between the Ser204-Ile216 constriction point (Fig. S3). Several angles are constant for long-lived conformations, yet highly flexible in the transition regions, especially between **B** and **C** (Fig. S4). The hinge pivot activity may be driven by backbone rearrangements necessary to accommodate hydrogen bonding interactions characteristic of conformations **B**–**D** (e.g., involving Pro 203, Ser204, and Ser212, Fig. S5) which are formed and broken over the course of the simulation. The contributions of hydrogen bonding to the conformation dynamics and lower event activity detected in regions populated by **B**–**D** is consistent with characterization of the Ω-loop as static^34,35^. The high variability of the hinge angles in the transition regions and the reversible **C**–**D** conformation change suggests **C** as a key loop conformation for Dio3 activity, which is supported by the locking of this conformation by T_4_ binding (vide infra).

### Structural features of the Ω-loop region

**Figure 3:**
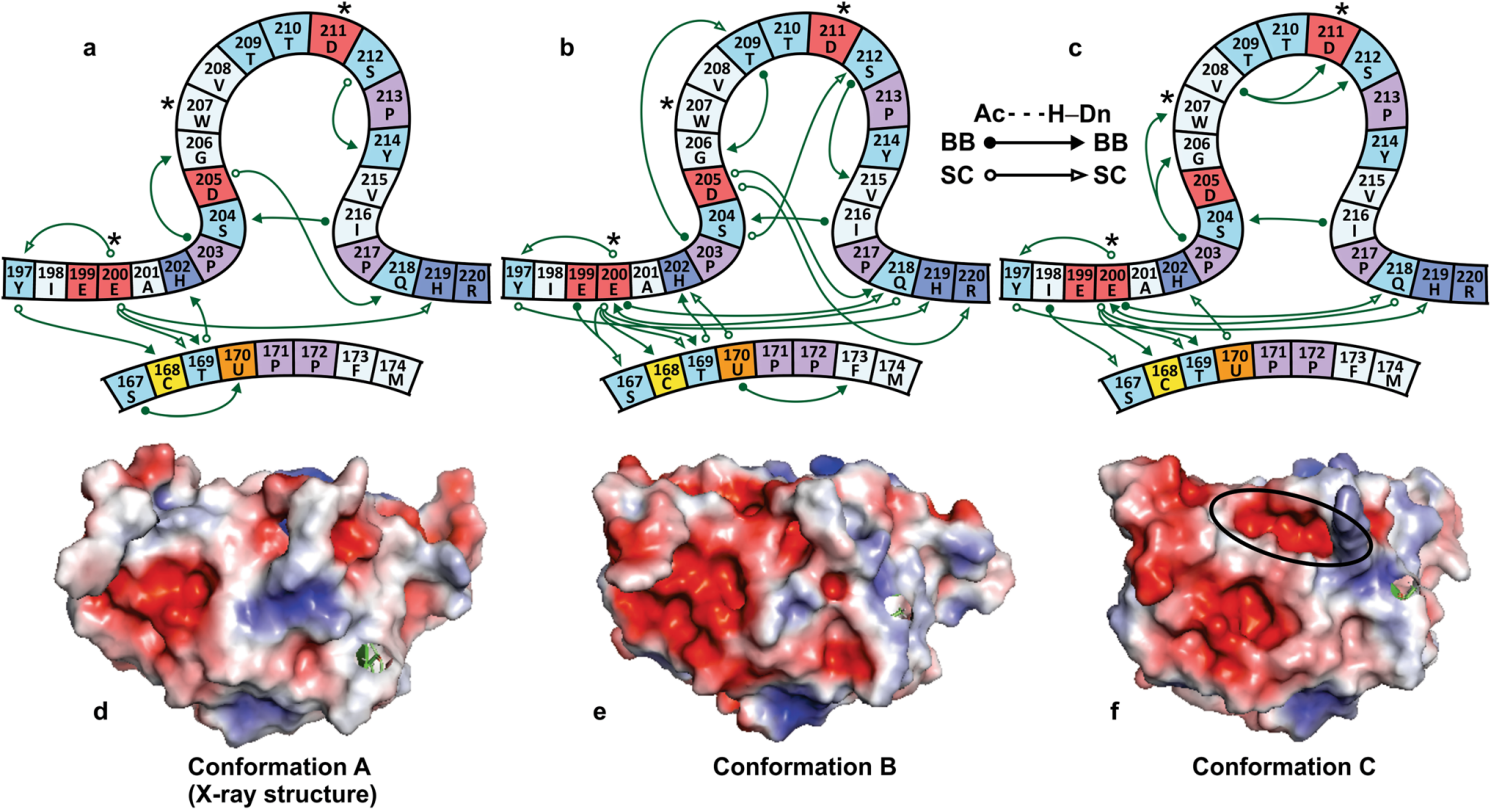
Diagrams of major hydrogen bonds between the Dio3 Ω-loop and the active site for conformations **A** (**a**, X-ray structure), **B** (**c**), and **C**, (**c**). Open (sidechain, SC) and closed (backbone, BB) circles and arrowheads indicate hydrogen bond acceptors and donors, respectively. Key residues Glu200, Trp207 and Asp211 that define the loop activity are indicated with (*). Hydrogen bonds to Glu200 anchor the N-terminal end of the loop to the active site SCTU motif. Minor interactions are not shown. A Ser204-Ile216 BB-BB interaction forms the constriction point of the loop. Changes in hydrogen bonding pairs within the middle loop sequence define the conformation. Interactions between the Trp207 indole and solvent contribute to loop instability. This residue folds into a hydrophobic pocket at conformation (**C**). Electrostatic maps of the X-ray structure (**d**) and conformations **B** (**e**) and **C** (**f**). Access to the Sec170 is blocked by Phe258 in (**A**). The pocket is partially occluded in (**B**). In (**C**), a cryptic pocket opens to the active site (indicated with an oval). Electrostatic maps created using PyMol 1.10.10.

Glu200 anchors the Ω-loop to the Dio3 active site (Fig. 3a) through an extended hydrogen bonding network that include conserved residues at the SCTU motif and C- and N-termini of the Ω-loop. Previously, Glu200 with His219 and Ser167 were proposed to participate in the proton cascade system that facilitates deiodination (Fig. 1c)^14,18,36^. In **A**, the extended Glu200 hydrogen bonding network links interactions between the Cys168 backbone (BB) NH and the sidechain (SC) of Tyr197, His202 NH_BB_ and Thr169 SC, and Arg225 NH_SC_ and His219 Nε (Fig. 3a). This latter interaction secures the loop C-terminus to α_D_. Most of these interactions remain largely intact throughout the simulation with the following changes and additions to the network maintained in **B** and **C** (Fig. 3). For example, the Tyr197 phenol shifts from Cys168 NH to interact with His219 NδH. Cys168 NH becomes part of the Glu200 anchor sharing the carboxylate with interactions to the Thr169 NH_BB_ and SC. The Gln218 SC also rotates to interact with the BB CO and NH of Glu200. Mutation of selected residues within this network significantly decreases experimental Dio activity (i.e., Glu200Ala, Thr169Ala, Ser167Ala)^14^.

Structural comparison of the principle Ω-loop conformations suggests that increasing exposure of polar sidechains to the solvent environment drives the initial conformation change from the X-ray structure **A** to **B**. The middle segment of the Ω-loop becomes pinched by several hydrogen bonds (i.e., Ser204-Ser212 SC-SC and Gly206-Thr209 BB-BB). A cross-loop SC-BB interaction in **A** between Asp205 and the Gln218 NH weakens as the Gln218 SC rotates to hydrogen bond with Glu200 BB CO. The Phe258 SC, which blocks access to Sec170 in **A**, rotates in and out of occlusion of Sec170 during the simulation. However, the shallow pocket to Sec170 is not well-suited for substrate binding as docking of T_4_ to **B** does not lead to a bound conformation.

**Figure 4:**
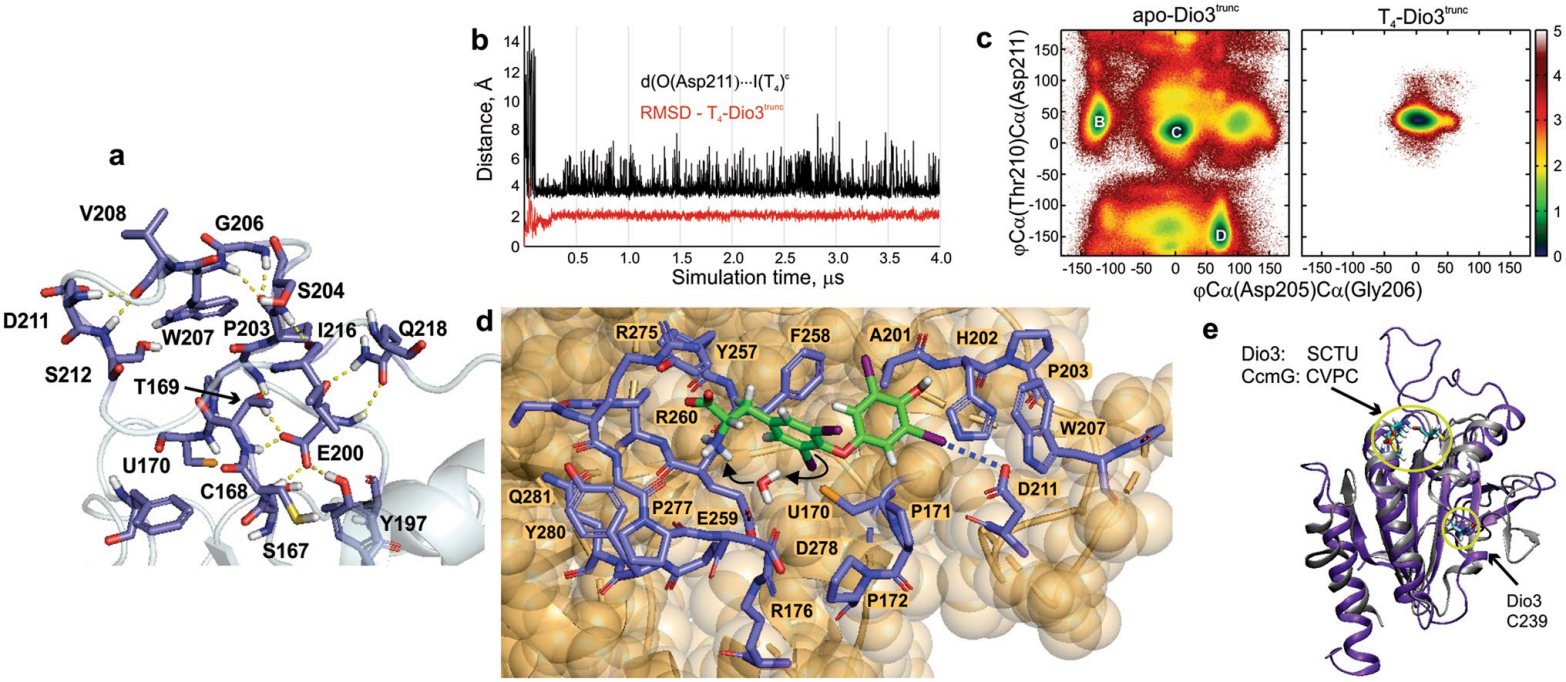
(**a**) Hydrogen bonding interactions within **C**. Glu200 anchors the loop to the active site to constrain its conformational space. Image created using PyMol 1.10.10. (**b**) Root-mean-square deviation (RMSD) of the T_4_-Dio3^trunc^ trajectory (red, loop residues only) indicates that the Ω-loop conformation is constant following a brief equilibration period. T_4_ is bound to the active site with the assistance of a secondary outer-ring I···O interaction with Asp211 (black) that reinforces regioselectivity of Dio3 for IRD. (**c**) Mapping of the conformation space of the Ω-loop in the apo-Dio3^trunc^ and T_4_-Dio3^trunc^ simulations. T_4_ binding significantly reduces the dynamics of the loop. (**d**) Thyroxine binding to the cryptic pocket of Dio3 through an I···Se XB interaction (enforced through the MM force field). Water molecules in the cryptic pocket facilitate protonation of the carbanion. Dummy atoms have been removed for clarity. Image created using PyMol 1.10.10. (**e**) Overlay of the X-ray structure of Dio3 (4TR4) and CcmG (1KNG). The CXXC motifs and the distant Cys239 of Dio3 are indicated. Image created using VMD 1.9.3.

Several key hydrogen bonding interactions that define **B** (i.e., Gly206–Thr209 BB–BB and Ser204–Ser212 SC–SC, Fig. S5 and Table S1) are lost after 2 μs as the loop undergoes an extended period of significant rearrangement. At 12 μs, the loop adopts the long-lived conformation **C** in which BB–BB bridging of the Asp211 and Ser212 NHs by Val208 CO replaces the Ser204–Ser212 SC–SC interaction characteristic of **B**. Similarly, the Pro203 CO spans the Gly206 and Trp207 NH_BB_s, limiting the mobility of the Gly206–Trp207 hinge pivot (Fig. S4). Most significantly, at **C** the Trp207 indole folds into a nonpolar cleft between Pro203 and Gly206 (Figs. 3c, 4a). Protrusion of Trp207 into the solvent is the primary contributor to conformational instability as indicated by the pair contact and hinge-pivoting fast motions (Fig. S3) and normal mode analysis (Fig. S7). Trp207 is conserved in all Dio types and its hydrophobic properties may contribute to substrate selectivity through control of the loop conformation. An Ω-loop Trp also contributes to the active site gating mechanism of acetylcholinesterase^37^. Conformation **C** undergoes a reversible change to **D** as the His202–Trp207 π-stacking interaction is lost and constriction point drifts to a Ser204–Val215 BB–BB interaction. **D** consists of two closely related conformations in which the active site is partially occluded by Tyr197 due to a loop C-terminus conformation change that moves His219 to the exterior of the protein.

### Formation of a cryptic pocket

Rearrangement to **C** coincides with the opening of a narrow cryptic pocket to Sec170 observable in the Dio3^trunc^ electrostatic potential (Fig. 3d–f). The pocket accommodates TH substrate binding through both an inner ring TH-Sec170 I⋯Se XB interaction and electrostatic interactions between the amino acid group and a polar cleft. Sec is partially buried in the pocket, protecting it from cellular oxidants. Phe258 forms a hydrophobic wall with Tyr257 and Ala201 near Sec170 (Fig. 4d). The reduced activity of the Phe258Pro and Tyr257Ala mutations^14^ could be attributed to conformational restrictions that prevent effective pocket formation. Arg176 salt-bridges Asp278 and Glu259 to form the opposite wall and floor of the pocket, respectively. Arg176 replacement by Lys and Asn in *mus* Dio1 and Dio2, respectively, may alter the pocket topology to affect selectivity. Pro171 and 172 abut the Arg176–Asp278 salt bridge and introduces a kink in α_1_ toward the Sec170 end of the pocket. On the same side, Gly276 and Pro277 interact with Arg275 for the wall near the polar cleft. The loop end of the pocket consists of a π-stacking interaction between His202 and Trp207, the latter of which remains tucked into the nonpolar Pro203–Gly206 cleft. The sidechains of Tyr257 and Arg275 face into the polar end of the pocket and are reinforced by Arg260 which also hydrogen bonds to the Tyr280 SC and Gly274 BB. Arg275 lies in the slow^34^, flexible β_4_α_3_ turn close to a proposed dimerization interface^14^ and opens and closes over this polar cleft as a possible gating mechanism for binding of the amino acid end of T_4_. Dimer formation at the α_3_β_4_ edge may dampen the motion of the turn contributing to a stronger interaction with the polar cleft relative to the monomer.

### Substrate binding to the cryptic pocket

Simulations were performed with T_4_ docked to the active site of conformations **A**–**D** assuming the formation of the initial inner-ring I⋯Se XB intermediate along the path to deiodination (Fig. 4)^18^. Dio3^trunc^-T_4_ complexes were equilibrated using a force field that treats the strong I⋯Se XB as a covalent bond with the XB charge distribution adapted from DFT model complexes^24^. All other iodines use charged dummy atom method of Jorgensen and Schyman^38^ to represent σ-holes^39^ along the C–I bond axes. This approach constrains T_4_ to the active site during the equilibration process while allowing weak intermittent electrostatic XB interactions between the ancillary iodines and the solvent or protein. Simulations with T_4_ docked to **A** (with Phe258 rotated to expose Sec170), **B**, or **D** did not result in stable Dio3^trunc^-T_4_ complexes because these conformations lack well-defined active site pockets. The cryptic pocket of **C** accommodates both the I⋯Se XB interaction and the T_4_ amino acid group in the cleft near Arg275 (Fig. 4d). Over a 4-μs simulation, the Ω-loop conformation is constant (Fig. 4b,c), and T_4_ remains bound to the pocket with minor structural changes, specifically a shift of the constriction point of the loop from Ser204–Ile216 BB–BB to SC–BB. Gln218 SC reinforces this shift through a hydrogen bond network with His202 BB and Ser204 SC. Hydrogen bonding interactions to the Arg275 and Tyr257 SC hold the T_4_ carboxylate in the cleft. The lack of a full salt bridge to the T_4_ carboxylate may contribute to the low activity of the Arg275Ala mutant^14^. Both Ala276 BB groups also stabilize the T_4_ amino acid group in the cleft.

Following a brief equilibration period, an I⋯O halogen bond forms between the outer-ring T_4_ iodine and Asp211 carboxylate with the loop locked in a slightly modified conformation **C** (Fig. 4b,c). The non-bonding I⋯O interaction is held in place by the electrostatic interaction between the Asp211 carboxylate and the positively charged dummy atom representing the outer-ring σ-hole of T_4_^38^. The other outer-ring iodine lies in a hydrophobic cleft created by the Ala201 and Phe258 sidechains and forms intermittent weak electrostatic XB interactions with solvent water.

The I⋯O interaction alternates between the oxygen centres of the sidechain with distances in the 3.0–3.5 range and ∠ C–I⋯O of between 165° and 180°, consistent with known I⋯O XB interactions (Figs. 4c, S6)^40,41^. The positioning of Asp211 is reinforced by hydrogen bonding to the Trp207 indole and a turn in the loop induced by a bifurcated BB-BB interaction between Val208 CO and Asp211 and Ser212 (Figs. 3b, 4a). Similar XB interactions are found for the interaction of Asp with the heme-bound 4-iodopyrzole in nitrophorin (3.30 Å, PDB 1ML7)^42^ and iodipamide in human serum albumin (3.48 Å, PDB 2BXN)^43^. The I⋯O interaction in the latter is supported by a Lys-Asp salt bridge similar to the Trp207-Asp211 interaction in Dio3^trunc^. Sulfation of the T_4_ phenol prevents Dio3 activity^44^, possibly due to inhibition of binding by repulsions with Asp211.

The presence of the secondary I⋯O XB interaction with the protein is consistent with the high affinity of Dio3 for T_3_, and its primary function of controlling plasma T_3_ levels through deactivation to 3,3′-T_2_^2^. Asp211 is conserved across species in Dio3, but not in Dio1 and Dio2. (Asp occurs one residue downstream in Dio1 for some species, but it is not conserved.) The secondary interaction also suggests a potential for lower Dio3 affinity for THs without outer-ring iodines (i.e., 3,5-T_2_ and 3-T_1_). PCBs or PBDEs having an *ortho* halogen on one ring and a *meta* on the other could be effective inhibitors of Dio3 due to their ability to reinforce the primary X⋯Se XB interaction with the secondary X⋯O interaction. Similar structural features may define the selectivity of Dio1 and Dio2, discovery of which may aid the design specific inhibitors for each Dio type.

His158 and His174 (analogous to His202 and His219 in Dio3, respectively) were shown to be important for *rattus* Dio1 activity^45^. Mutation of His158 in Dio1 results in a complete loss of activity. His202 was proposed to act with Arg275 to clamp TH into the active site by a hydrogen-bonding interaction with the 4′-OH group in analogy to the T_3_-bound receptor β^14,46^. In the current simulations, protonated His202 is sandwiched between the T_4_ outer ring and Sec170. Simulations of His202 in the deprotonated state show no interaction with the phenol and loss of T_4_ binding to the pocket. His174 is conserved for *mus* Dio1-3, but Arg275 in Dio3 is not. Dio1 has Arg one residue earlier in its sequence than Dio3 and Dio2 replaces Arg with Lys. These observations could suggest a cooperative interaction between His and Arg/Lys that, along with the differing Ω-loop sequences in Dio1-3, may contribute to regioselectivity. Alternatively, His202 may have a different role in Dio activity, such as enhancing Sec170 nucleophilicity deprotonation^47^. Simulations indicate that His219 in Dio3^trunc^ plays a structural role through an extended hydrogen bonding network with Arg225 and Tyr197. Loss of Arg225-His219 interaction in region **D** contributes to the collapse of the cryptic pocket which agrees with the proposed importance of its Dio1 analogue His174 for substrate binding, rather than catalysis^45^. Mutation to Gln or Asn, which can maintain similar hydrogen bonding interactions to His, increase K_M_ with no effect on V_max_^45^.

MD simulations of the T_4_-Dio3^trunc^ complex indicate that water molecules in the binding pocket likely to act as proton donors for the carbanion formed by deiodination. These waters lie primarily on the Arg176 side of the cryptic pocket and bridge the −NH_3_^+^ group of the T_4_ amino to the carbon of the inner-ring C−I bond activated by the I⋯Se XB (Fig. 4d). Several other water molecules seep into the cryptic pocket to hydrate the selenolate of Sec170. A water also bridges the sidechains of Ser167 and Thr169. Based upon the X-ray structure, Ser167 was assigned as the proton donor for nucleophilic deiodination and mutation to Ala reduces activity by > 90%^14^. However, in simulations, this residue lies on the opposite side of the Phe258-containing hydrophobic wall of the cryptic pocket from T_4_ where its alcohol group cannot contact the substrate.

Mutations to Ω-loops are known to destabilize protein structure^28^. The formally disordered Dio3 Ω-loop contributes to substrate selectivity through rigid conformations that position Asp211 for stabilization of T_4_ binding and enhanced selectivity for IRD. Mutations of the residues responsible for these rigid conformations will result in loss of the required conformation for TH binding and reduced activity. Several Dio3 mutants (Glu200Thr, Tyr197Phe and Thr169Ala^14^) in the region that anchors the C-terminus of the Ω-loop to the active site completely deactivate the protein. Although Glu200 was proposed to participate in the proton exchange network critical for catalytic deiodination^14,18^, in simulations it serves as the central residue of the conserved anchor region, forming interactions with Thr169 and Tyr197 that limit the flexibility of the Ω-loop. Mutation of the analogous Glu in human Dio1-3 to Ala results in inactive proteins^15^ because the unconstrained loop cannot form conformations necessary for substrate binding. In contrast, Glu200Asp maintains the ability to form this central hydrogen bonding network and either increases K_M_ or has no effect on activity^14^. A Thr169Ser mutation reduces the activity by only 60% because the protein can maintain the connectivity of the Glu200 anchor region^14^. The void created by the missing methyl group impacts the packing of hydrophobic sidechains (i.e. Ile216) in the loop and alters the orientation of Asp211 for a negative effect on substrate binding.

## Discussion

Regeneration of the selenolate by reduction of the selenenyl iodine is presumed to proceed through a selenosulfide intermediate. Internal Sec170-Cys239 selenosulfide bond formation was proposed^14^ in analogy to PtGPX5, an atypical 2-Cys peroxiredoxin (Prx), where a Cys44-Cys92 disulfide bond is observed in the oxidized protein (PDB: 2P5R^48^). The peroxidic Sec170 and Cys44 residues are similarly located in Dio3 and PtGPX5 (BLAST sequence similarity 17%), respectively. However, Cys92 is located in the equivalent of α_D_, where Cys239 is downstream in the α_D_β_D_ loop. Both residues are distant from the active site (22.3 and 16.0 Å, resp.). Cys44-Cys92 disulfide bond formation in PtGPX5 requires unravelling of the α_D_ helix facilitated by repulsions between Cys92 and two acidic residues, Asp85 and Asp89, positioned at the helix turns^48^. A similarly-located unstable helix without destabilizing acidic residues in *E. coli* thiol peroxidase (PDB: 3HVV) positions the resolving Cys on the interior of the protein closer (~ 11.5 Å) to the peroxidic Cys^49^. In contrast, Dio3 α_D_ lacks residues to induce helix-coil transitions and its residues do not fluctuate significantly in simulations, although the α_D_β_D_ turn itself is moderately flexible (Figs. S1, S3).

In Dio1 and Dio3, the upstream Cys of the SCTU motif may resolve the selenenyl iodide by a similar mechanism to active site CXXC motifs in redox Trx-fold proteins^17^. For example, cytochrome maturation proteins and thiol peroxidases, which are structurally similar to Prx and Dio, have CXXC motifs that form cyclic disulfides (i.e., PDB 1KNG^50^, Fig. 4e)^51^. The VCTU motif at the β_1_α_1_ turn of *Drosophila* Sep15 (PDB: 2A4H), a Trx-fold selenoprotein, also contains a disulfide bond in the Cys mutant^52^. In Dio3, both Cys lie within a channel with access to Sec170, but Cys168 is closer to Sec170 (in **C**, d(Se_Sec_⋯S_Cys_) ≈ 8 Å compared to 17 Å for Cys239), nucleophilic attack by Cys168 would require minimal rearrangement of the β_1_–α_1_ loop, and the greater reactivity of Sec favors reaction on a shorter timescale than helix unfolding. Studies show lower activity for the Cys168Ala mutant^14^, but no change for Cys239Ala, suggesting the former is more critical for turnover. Dio2, which is missing the active site Cys yet retains the analog of Cys239, deiodinates one equivalent of T_4_ and is tagged for destruction by ubiquitin^53,54^. The SATU motif in Dio2 may trap Sec in an intermediate susceptible to ubiquitin tagging that cannot be resolved without the assistance of the active site Cys. Alternatively, Cys239 could be positioned to attack the Cys168 end of the selenosulfide to release the selenolate and form a Cys168-Cy239 bond as observed in PtGPX5. Similarly, Cys73 in PtGPX5 has a shorter pathway to Cys44 (d(S⋯S) = 11.5 Å versus 22.3 Å for Cys92) and would require less rearrangement to attack Cys44. An initial Cys44-73 disulfide bond could induce the helix–coil transition with subsequent thiol-disulfide exchange to Cys44-Cys92. Regardless of which Cys in Dio3 is resolving, the large distances to Sec170 preclude a deiodination mechanism assisted by a Se⋯S chalcogen bonding interaction as proposed for naphthalene-based Dio mimics^21^.

The two residues following Sec appear to define the overall kinetics of deiodination. A Pro-Pro sequence in Dio2 and Dio3 correspond to the ping-pong kinetics, whereas Pro-Ser in Dio1 corresponds to sequential kinetics and inhibition by propylthiouracil (PTU)^14^. Mutation of Pro135 in Dio2, analogous to Pro172, to Ser changes the enzyme kinetics of deiodination from ping-pong to sequential^15^. Mutation of the Pro-Pro sequence to Pro-Ser allows PTU to target Dio2 and Dio3. Dio1 in some fish species contain the PP sequence and are also PTU insensitive^7^. The second residue may be important for rearrangements needed to resolve the -SeI intermediate^14^. Because PTU is unreactive toward selenium functional groups in their most reduced states, Sec170 must first be formally oxidized by deiodination of T_4_. It is not clear whether PTU attacks the selenenyl iodide or the selenosulfide. In the selenenyl iodide state, Sec170 is protected by the bulk of the protein and the rigidity of the Pro-Pro sequence. Rapid resolution of -SeI by Cys168 releases iodide such that PTU could access Sec170 by the cryptic pocket. The Pro-Pro sequence may block Sec170 where Pro-Ser could induce a conformation change with an open pocket.

Experimental study of substrate binding to the iodothyronine deiodinase (Dio) family is challenging because these membrane-bound selenoproteins are difficult to isolate. Molecular dynamics simulations of the Type III Dio (Dio3) monomer provide atomistic insight into the possible mechanism for substrate binding and regioselectivity for the activation and control of the cellular thyroid hormone concentration. Thyroxine binding is found to depend upon the conformation of a Dio-specific Ω-loop and the opening of a cryptic pocket. This flexible loop is adjacent to Sec170 and rearranges from the X-ray structure to minimize solvent interactions with nonpolar residues. Multi-microsecond simulations were required to fold the conserved Trp207 into a hydrophobic cleft to populate a key loop conformation. Opening of a cryptic pocket in conjunction with this loop conformation allows thyroxine binding through a polar cleft for the amino acid group and an I⋯Se inner-ring halogen bond to Sec170. Thyroxine binding to the pocket is stabilized by an I⋯O halogen bonding interaction between the σ-hole of an outer ring iodine and the Asp211 carboxylate. Protonation of the carbanion leaving group during deiodination is facilitated by water molecules in the pocket.

Additional features identified by MD simulations provide an alternate perspective on the overall Dio3 mechanism. The location of Cys168 in the SCTU motif is consistent with a backside attack on the selenenyl iodide to a cyclic selenosulfide found in the X-ray structures of oxidized Trx-fold proteins such as CcmG and Cys mutant of the Sep15 selenoprotein. Cys239 is too distant from Sec170 to directly resolve the selenenyl iodide. The α_D_ helix near Cys239 is rigid and lacks destabilizing residues that induce unravelling of the analogous helix in PtGPX5.

The residues that make up the anchor region around Glu200, specifically Ser167, Thr169, and residues at the N- and C-termini of the loop are highly conserved across the same Dio types with significant variation in the middle sequence of the Ω-loop. Hydrogen bonding around Glu200 fixes the early sequence of the loop to the active site and limits its conformation dynamics. Previous experimental mutation studies of residues involved in this anchor region lead to inactive proteins. Disruption of hydrogen bonds anchored at Glu200 by mutation of key residues (e.g., Ser167Ala, Glu200Ala, Thr169Ala) likely allow too much flexibility for the formation of the key loop conformation which prevents substrate binding or alignment of a previously proposed proton transfer pathway.

The highly conserved Trp207 appears to drive the conformation dynamics of the Ω-loop. Folding of Trp207 into a nonpolar cleft correctly positions the loop to reinforce the I⋯O halogen bond between an outer-ring TH iodine and the carboxylate side chain of Asp211. This residue is conserved only in Dio3 where it is required for the IRD regioselectivity of Dio3. The sequence and length of the Ω-loop between Trp207 and His219 varies significantly between Dio1-3, but with high similarity within types. These variations likely contribute to regioselectivity by allowing type-specific conformations capable of orienting a TH for IRD or ORD. The conserved Trp207 may control loop conformation for regioselective deiodination. Given that the interpretations of the MD simulations of the apo- and T_4_-Dio3 proteins are consistent with previous mutation studies of key residues (e.g., Glu200, His219, etc.), new mutation studies may confirm the importance of Trp207 and Asp211. Additionally, comparative MD simulations of Dio1 and Dio2 will determine how the conformational preferences of different loop sequences contribute to regioselective TH binding, which may lead to the design of inhibitors tailored to specific Dio types to treat TH-related illnesses.

## Methods

The protein model was modified from the X-ray structure of the catalytic region of *mus* Dio3 (PDB: 4TR4; residues 114–301) by removing the linker region (residues Gly114 to Gly131) to the membrane-bound domain and capping the N-terminus with an acetyl group (Dio3^trunc^). Hydrogens were added to the X-ray structure using the H++ server^55^. Dio3^trunc^ was modelled as the monomer because the homodimer structure has not been characterized experimentally. Alternate possible dimerization interfaces have been proposed^14,56^. In simulations of the full 4TR4 protein, the N-terminal linker sequence wrapped around the protein to interact with the Ω-loop. This tendency may contribute to the experimental inactivity of the monomeric catalytic region^14^ if representative of the solution behaviour of the protein. Sec170, located in the α_1_β_1_ turn and facing a region bounded by the β_2_α_D_, α_2_β_3_, and β_4_α_3_ loops, is deprotonated at physiological pH^57^. The model assumes that its proton is transferred to nearby His202 to increase Sec nucleophilicity as observed in other selenoproteins^47^.

Normal mode analysis was performed using the anisotropic and Gaussian network models (ANM and GNM, respectively) within the ProDy open-source package (see Supporting Information)^58^ MD simulations were performed using the PMEDA GPU routines in AMBER 16^59^. The protein was represented with the *ff99sb* force field. Parameters for Sec were modified from the deprotonated Cys force field using charges, bond distances and force constants derived from DFT calculations. T_4_ was roughly docked to conformations **A**–**C** using AutoDock Vina^60^. The T_4_ force field was derived using ANTECHAMBER. The T_4_-Dio3^trunc^ model assumes a strong I⋯Se XB interaction between an inner-ring iodine and the Sec residue dominated by bonding character^18,19^. Charges for the XB T_4_ and the I⋯Se and force constant were obtained from DFT calculations of the T_4_–MeSe^−^ complex^24^. The remaining iodines use the method of Jorgensen and Schyman to mimic the σ-hole using a positively charged dummy atom^38^. The protein models were solvated with a 10 Å octahedral box of TIP3P water. Bond lengths to hydrogen atoms were constrained using SHAKE. Systems were initially warmed and equilibrated to 300 K using Langevin dynamics in the NVT ensemble. Systems were then equilibrated in the NPT ensemble prior to multi-μs production simulations. Statistical analysis of the trajectories is described in Supporting Information^32,33^. Electrostatic surfaces were generated by the Adaptive Poisson–Boltzman Solver within the PyMol graphical user interface (version 1.10.10)^61^. Additional images were created using VMD version 1.9.3^62^.

The apo-Dio3^trunc^ trajectory was stripped of solvent and hydrogens for processing using the TimeScapes package of python routines for statistical analysis of MD trajectories^32,33,63^. This methodology differs from the traditional RMSD (Fig S1a) in that a course-grained model is imposed on the trajectory which is then analysed in terms of side chain motions rather than Cα. Rates of pairwise contact and hinge-pivot events were determined based upon (a) cutoff distances and (b) generalized masked Delaunay (GMD) tetrahedralization of representative atoms of the course-grained protein and counted subject to the requirement of crossing a buffer region defined either in terms of a cutoff distance or the GMD order. The dependence on pairwise contacts and backbone motions was determined using both Pearson cross-correlation and mutual information approaches. The Dio3^trunc^ trajectory was culled to 2000 snapshots by extracting frames at 100 ns intervals. The RMS fluctuation of the Cartesian coordinates was first determined using a sliding window of 10 frames with least-squares fitting to the X-ray structure. Analysis of the Dio3^trunc^ trajectory applied a cutoff buffer of 6.0–8.5 Å and a GMD buffer between graphs of orders 2 and 3 (Fig. S2).

## Supplementary information

Supplementary Information.
