# Supplementary material for: Thyroxine binding to type III iodothyronine deiodinase
Source: Sci Rep. 2020 Sep 21;10:15401. doi: 10.1038/s41598-020-72243-9 (PMC7506546; doi:10.1038/s41598-020-72243-9)
Supplement: Supplementary file 1 — Supplementary Information. [file 41598_2020_72243_MOESM1_ESM.docx]

**Supplementary Materials for:**

**Thyroxine binding to Type III iodothyronine deiodinase**

Craig A. Bayse*, Eric S. Marsan, Jenna R. Garcia, and Alexis Tran-Thompson

email: cbayse@odu.edu

*Department of Chemistry & Biochemistry*

*Old Dominion University*

*Norfolk, VA 23518*

*USA*

**1. Additional details for apo- and T_4_-Dio3^trunc^ MD simulations**

The trajectory of the Dio3^trunc^ model was sampled over 20 μs to explore the highly flexible nature of the Ω-loop which initially rearranges from the X-ray structure conformation (Fig. S1a). Examination of the traditional root-mean-square deviation (RMSD) calculated from the Cα positions of the loop reveals that an initially formed stable conformation **B** is lost within 5 μs followed by ~5 μs of instability before the loop settles on conformation **C**. The integrity of this conformation is lost for ~5 μs (**D**) before **C** is revisited and stable for several μs until the end of the simulation. The RMSF (Fig. S1b) is consistent with statistical analysis of the trajectory, normal mode analysis, and the experimental X-ray structure B-factors (*vide infra*). Structures in PDB format can be requested by emailing [cbayse@odu.edu](mailto:cbayse@odu.edu).

**
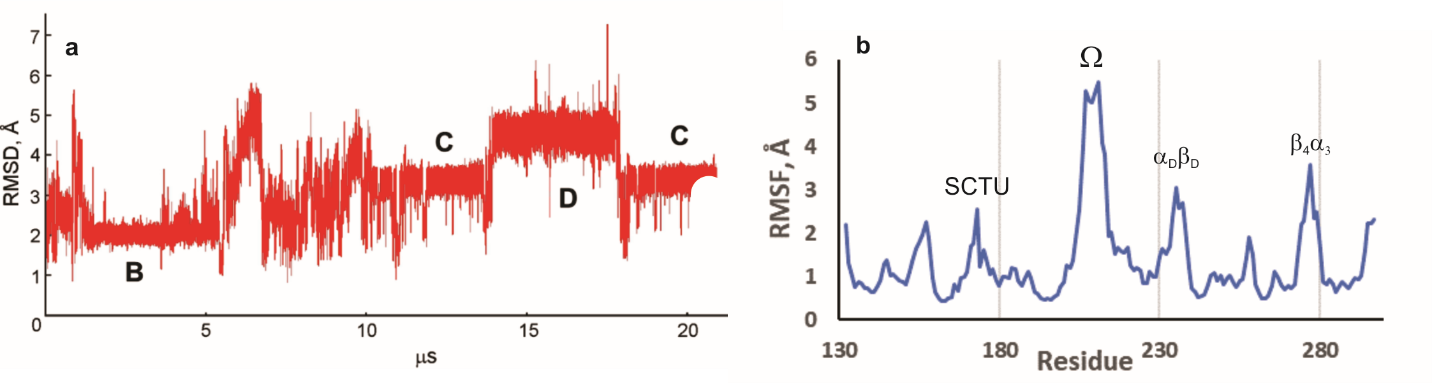
**

**Figure S1.** (a) Root-mean-square deviation (RMSD) for the Ω-loop of *mus* Dio3^trunc^. The X-ray structure (**A**) is the reference structure. (b) Root-mean-square fluctuation (RMSF) of Cα for the simulation of apo-Dio3^trunc^.


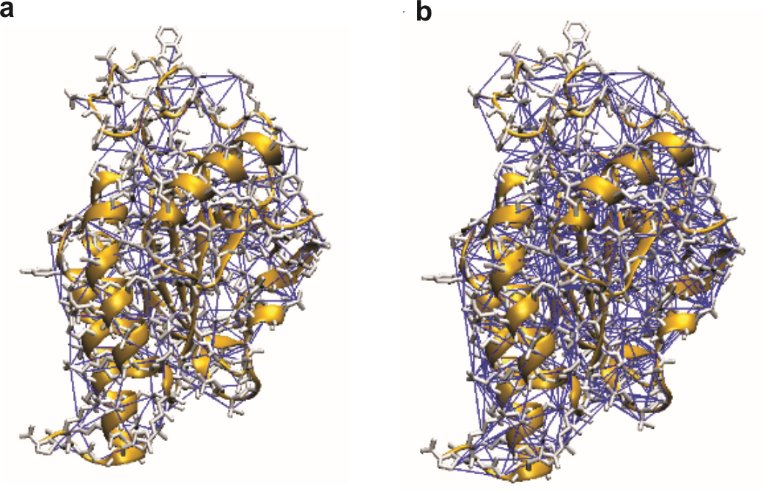


**Figure S2**. GMD side chain tetrahedralization for Dio3^trunc^ is shown for the X-ray structure **A**: (b) order 2 and (c) order 3. These graphs were used to define the buffer boundaries for the GMD-based activity analysis of the apo-Dio3^trunc^ trajectory. Image created using TimeScapes version 1.5 ^1^ and VMD version 1.9.3.^2^


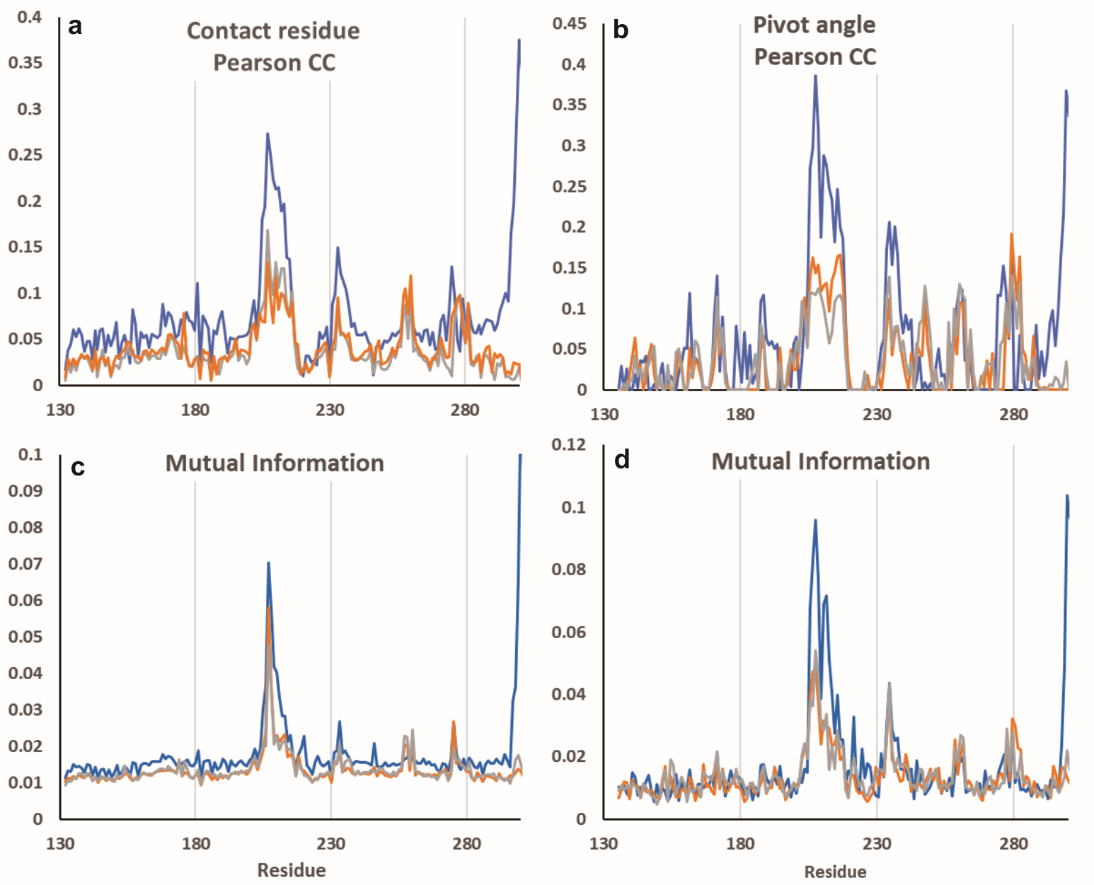


**Figure S3.** (a,c) Contact residue profile calculated using the Pearson cross correlation (CC) and mutual information (MI) methods, respectively.^3,4^ (b,d) Pivot angle profiles calculated using the CC and MI methods.


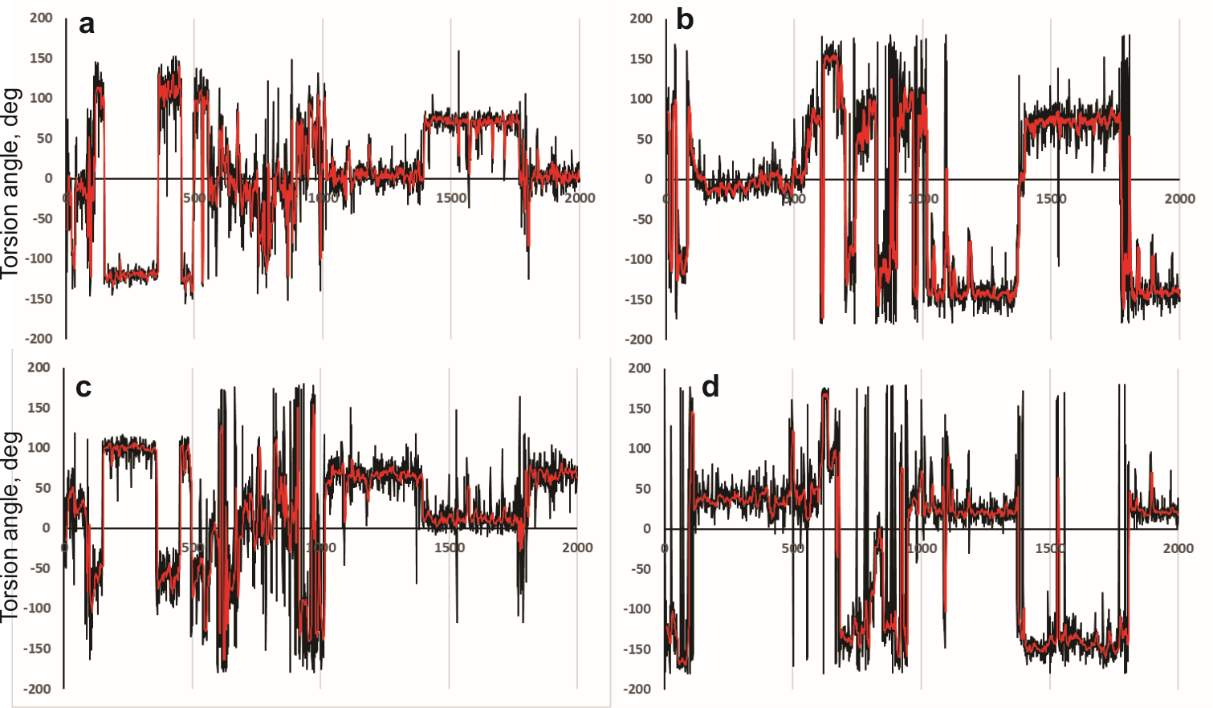


**Figure S4.** Torsion angle with significant contribution to the Ω-loop conformation dynamics. Dependence of the rotation around the following bonds (black line, running median – red line) (a) Cα(Asp205)−Cα(Gly206) (b) Cα(Trp207)−Cα(Val208) (c) Cα(Gly206)−Cα(Trp207) (d) Cα(Thr209)−Cα(Asp211)

Hydrogen bonding interactions that define the Ω-loop conformation were omitted from the statistical analysis of the trajectory. Specific pairs of hydrogen bond donors/acceptors are highly populated in conformations **B**-**D** (see Fig 2 in the main text and Table S1). These observations are consistent with the static characterization of the loop in section 3 (*vide infra*). The hinge pivot activity can be viewed as driven by the rearrangements of the loop backbone necessary to accommodate key hydrogen bonding interactions formed and broken over the course of the simulation. Several examples of characteristic hydrogen bonds involving Pro 203, Ser204, and Ser212 and their evolution over the simulation are shown in Fig S5.

**Table S1**. Hydrogen bond populations over the full simulation and the regions populated by conformations **B**-**D**. SC = side chain group; BB = backbone group; * = population less than 1%.

| **H bond acceptor** | **H-bond donor** | **Full** | **B** | **C** | **D** |
| --- | --- | --- | --- | --- | --- |
| **Anchor Region** | |  |  |  |  |
| Glu200 SC | Tyr197 SC | 90.0% | 99.8% | 99.7% | 59.0% |
| Tyr197 SC | Cys168 BB | 7.9% | 2.0% | * | * |
| Tyr197 SC | His219 SC | 37.5% | 72.3% | 10.3% | * |
| Glu200 SC | Thr169 SC | 99.9% | 99.9% | 99.9% | 99.9% |
| Glu200 SC | Thr169 BB | 97.2% | 98.5% | 99.0% | 98.5% |
| Thr169 SC | His202 BB | 28.8% | 46.8% | 1.1% | 2.6% |
| Glu200 SC | His219 SC | 6.6% | 1.8% | * | * |
| Glu200 SC | Ser167 SC | 4.7% | 1.8% | * | 6.5% |
| Ile198 BB | Ser167 SC | 40.0% | 12.0% | 80.4% | 79.8% |
| Glu200 SC | Cys168 BB | 85.4% | 84.4% | 98.6% | 96.9% |
| Glu200 SC | Gln218 SC | 59.7% | 90.4% | 28.0% | 60.4% |
| Glu199 BB | Ser167 SC | 33.8% | 72.3% | * | 1.9% |
| Sec70 SC | His202 SC | 67.3% | 61.0% | 87.0% | 90.5% |
| **Ω-loop sequence** | |  |  |  |  |
| Pro203 BB | Gly206 BB | 21.9% | * | 80.1% | 6.1% |
| Pro203 BB | Thr209 SC | * | 41.1% | * | * |
| Pro203 BB | Trp207 BB | 15.2% | * | 58.5% | 4.1% |
| Ser204 BB | Val208 BB | 15.0% | * | * | 63.3% |
| Ser204 BB | Ser212 SC | 17.6% | 96.9% | * | * |
| Asp205 SC | Asn218 BB | 13.2% | 69.7% | * | 1.2% |
| Asp205 SC | Thr210 SC | 23.57% | 85.6% | * | * |
| Val208 BB | Asp211 BB | 21.3% | * | 78.2% | 5.4% |
| Val208 BB | Ser212 BB | 21.8% | * | 82.8% | 4.8% |
| Thr209 BB | Gly206 BB | 13.5% | 86.0% | * | * |
| Ser212 SC | Tyr214 BB | 19.1% | 3.5% | 1.6% | 32.4% |
| Ser212 BB | Asp205 BB | 34.1% | 86.0% | * | 34.1% |
| Ser212 BB | Gly206 BB | 14.2% | 2.7% | * | 60.3% |
| Ile216 BB | Ser204 BB | 71.6% | 86.6% | 89.1% | 11.5% |
| Val215 BB | Asp205 BB | 9.8% | * | * | 41.3% |

**
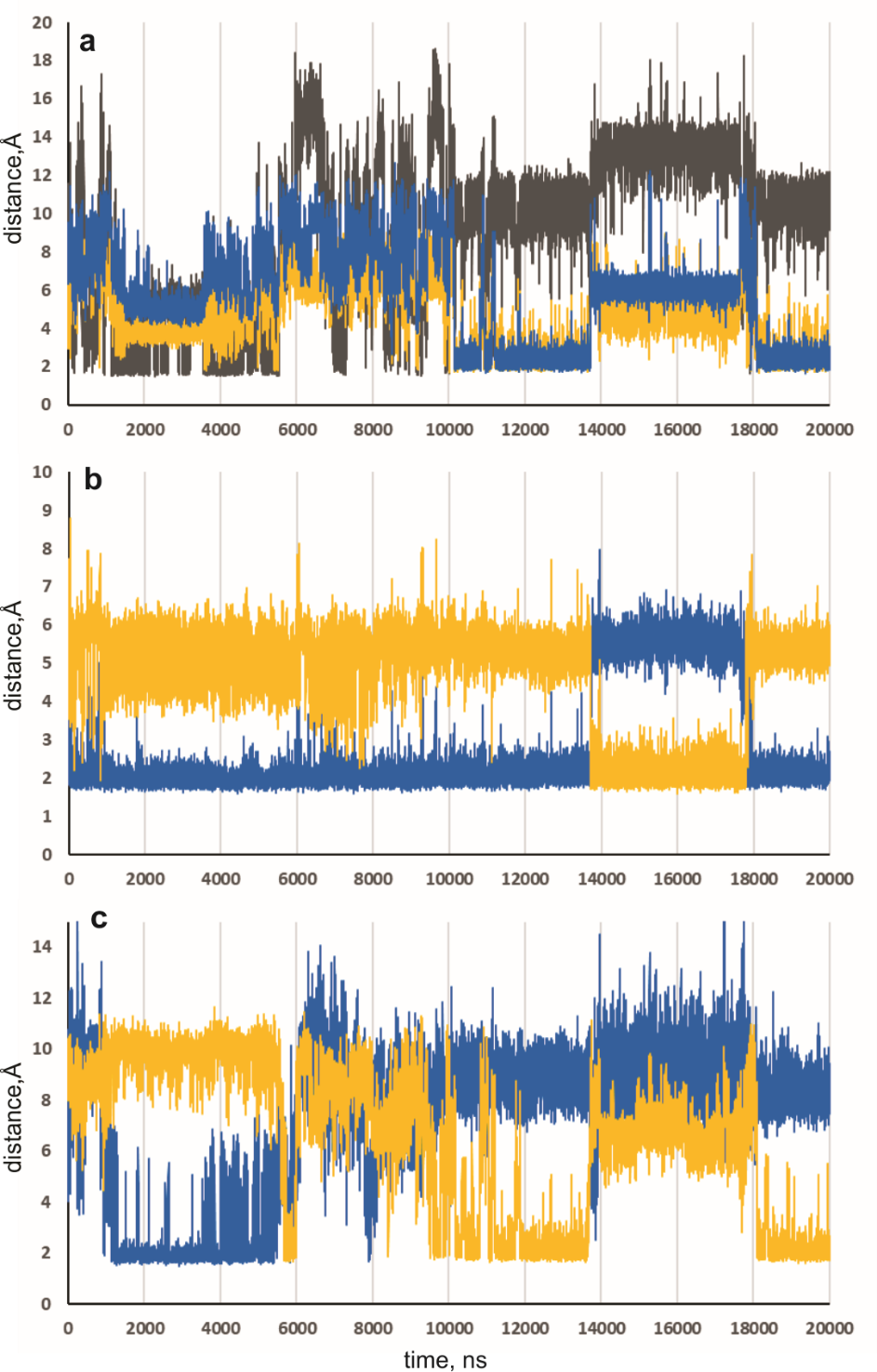
**

**Figure S5**. Comparison of selected distance measurements for hydrogen bonds that characterize Ω-loop conformations (Fig 3): (a) the Pro203-Gly206 BB-BB interaction (orange) is found within **A**, but initially lost as the protein relaxed in solvent in favor of a Pro203 CO BB interaction with the Thr209 SC (grey) in **B**. The Pro203-Gly206 interaction reforms at **C** but Pro203 CO also bridges the Trp207 BB NH (blue) due to the change in loop conformation. (b) The Ser204-Ile216 BB-BB (orange) interaction at the Ω-loop constriction point is temporarily replaced by a Ser204-Val215 BB-SC (blue) interaction in region **D**. (c) The Ser204-Ser212 SC-SC (blue) interaction populated in **B** is replaced by interactions with solvent and a Val208-Ser212 BB-BB interaction (orange).

**
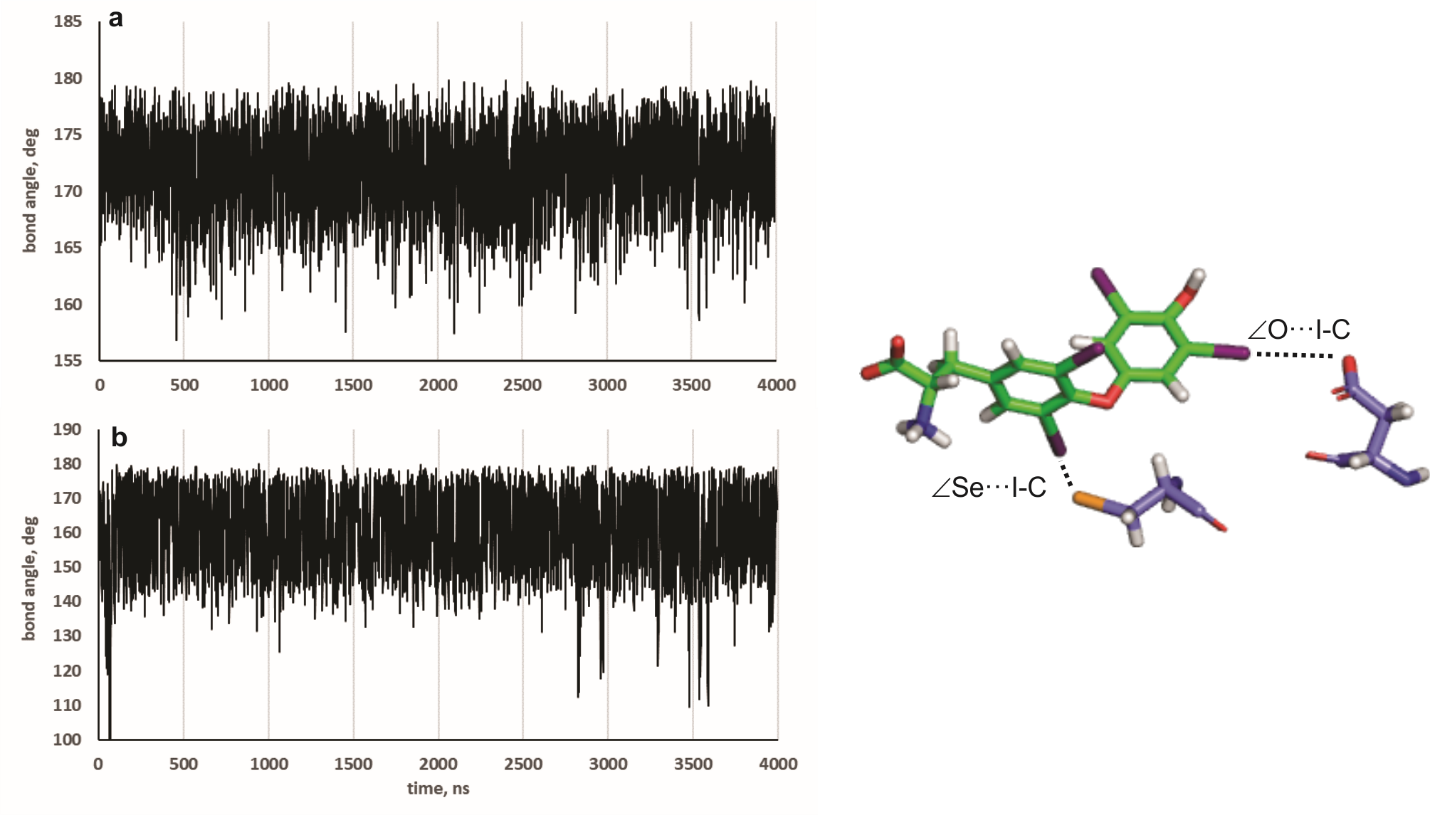
**

**Figure S6.** Bond angles for halogen bonding interactions for the T_4_-Dio3^trunc^ simulation: (a) ∠Se−I−C for the inner-ring bond-like I⋅⋅⋅Se XB; (b) ∠O−I−C for the I⋅⋅⋅O halogen bond facilitated using the Jorgensen-Schyman XB force field in which the σ-hole is represented by a charged dummy atom along the C-I bond axis. Inset image creating using PyMol version 2.3.2.^5^

**2. Normal Mode Analysis**

Normal mode analysis using elastic network models (ENMs) can predict the mobility of protein sequences without expensive MD simulations. These methods are most accurate near the global minimum and can be compared with B-factors from X-ray crystallography. Normal mode analysis was performed on the truncated Dio3 X-ray structure (PDB: 4TR4) as processed by H++ using the anisotropic and Gaussian network models (ANM and GNM, respectively) within the ProDy open-source package.^6^ Each method predicts high mobility in the Ω-loop, but the GNM provides the best agreement with the experimental B-factors with atomic motion predicted at the N- and C-termini, β_N1_β_N2_ turn, θ_1­_β_1_ turn, α_D_β_D_ turn (including portions of the adjoining helix and strand), and β_4_α_3_ turn (including portions of the adjoining helix), albeit with significantly lower intensity relative to the Ω-loop compared to the experimental values (Fig. S7). These regions of flexibility agree with the root-mean-square fluctuations (RMSF) of the α-carbons determined from the apo Dio3 simulation (Fig. S1a) and the per-residue activity profiles (Fig S3). Recalculation of the normal modes at conformation **C** produces similar mobility of the Ω-loop and the C-terminal, but more localized to the turn sequences and missing contributions from the α_D_β_D_ turn suggesting that relaxation within the solvent model increases interactions that stabilize this turn. Examination of the first 10 normal modes for Dio3^trunc^ at the X-ray conformation **A** (with the flexible N-terminal linker region removed) indicate that the slow motions are dominated by the Ω-loop and the C-terminal tail of α_3_ with minor contributions from the α_D_β_D_ and β_4_α_3_ turns, containing Cys239 and Arg275, respectively. The slowest modes are dominated by the motions of the C-terminus and Ω-loop. Trp207 makes the most significant contribution to mobility consistent with its key role in driving the conformation dynamics of the Ω-loop. Conservation of this residue throughout the Dio family is consistent with driving the intrinsic flexibility of the loop. The correlation of the normal modes (Fig S8) indicates that the motion of the active site SCTU sequence and the Ω-loop are only slight correlated using ANM, but more so in GNM. Residues of the α_D_β_D_ loop containing Cys239 is also correlated with the active site, which could support this residue as the resolving Cys, but the rigidity of the adjoining helix and strand disfavors this assignment.


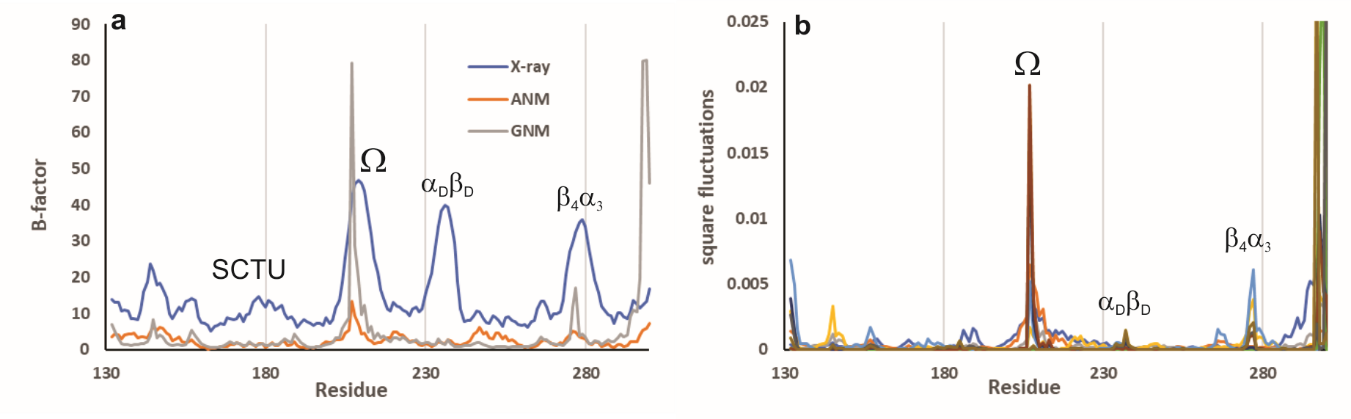


**Figure S7.** Normal mode analysis of Dio3. (a) Comparison of the B-factors of the X-ray structure of *mus* DIO3 (PDB: 4TR4) to the predicted values using ANM and GNM. (b) By-residue Cα displacements for the first 10 slow modes calculated using GNM.


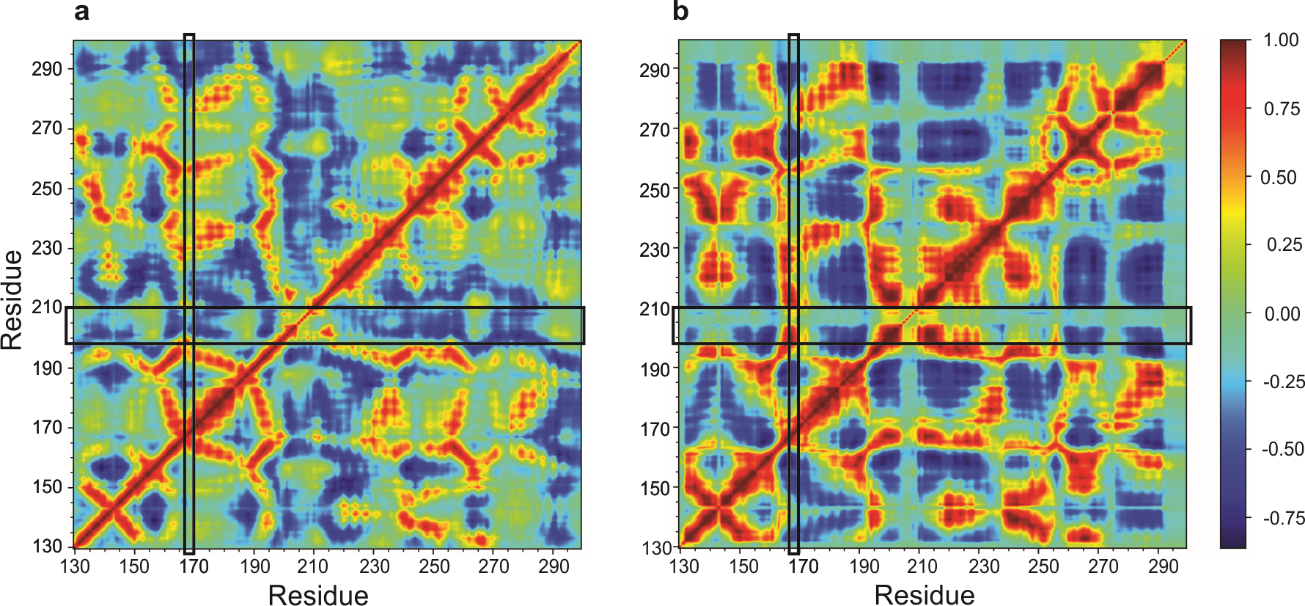


**Figure S8**. Cross correlation of residue motions within normal mode analysis of Dio3^trunc^ at the X-ray structure conformation **A** calculated using (a) ANM and (b) GNM. Image created using ProDy version 1.10.10.^6^

**3. Characterization of loop mobility**

The Dio3^trunc^ structure was processed through the Brüschweiler group’s statistical database for loop mobility (<http://spin.ccic.osu.edu/index.php/toeloopppi>).^7,8^ The Ω-loop sequence between the constriction residues (Ser204-Ile216) includes a high fraction of residues preferentially found within static (Tyr214, Ile216) and slow loops (Trp207, Val208, Thr209, Thr210, Val215). Only two residues are common to fast loops (Asp205, Asp211). Processing of the H++ processed X-ray structure **A** classifies the Ω-loop sequence as static, which is consistent with hydrogen bonding interactions involving Thr and Trp residues and relative long lifetimes of the persistent conformations **B**-**C** found in MD. In contrast, the α_D_β_D_ loop near Cys239 is characterized as fast in analyses of conformations **A** and **B**, but static in configurations **C** and **D** due to rearrangements of the structure within the solvent environment. The β_4_α_3_ turn containing Arg275 is slow consistent with a possible gating mechanism for binding of the amino acid end of T_4_ in the polar cleft of the active site pocket.

**References**

1. Wriggers, W. *TimeScapes Analytics Package*. <http://timescapes.biomachina.org/> (2017).

2. Humphrey, W., Dalke, A. & Schulten, K. VMD: Visual molecular dynamics. *J. Mol. Graph.* **14**, 33–38 (1996).

3. Wriggers, W. *et al.* Automated Event Detection and Activity Monitoring in Long Molecular Dynamics Simulations. *J. Chem. Theory Comput.* **5**, 2595–2605 (2009).

4. Kovacs, J. A. & Wriggers, W. Spatial Heat Maps from Fast Information Matching of Fast and Slow Degrees of Freedom: Application to Molecular Dynamics Simulations. *J. Phys. Chem. B* **120**, 8473–8484 (2016).

5. DeLano, W.L. *PyMol Molecular Graphics System*. https://pymol.org/ (Schrodinger, Inc., 2015).

6. Bakan, A., Meireles, L. M. & Bahar, I. ProDy: Protein Dynamics Inferred from Theory and Experiments. *Bioinformatics* **27**, 1575–1577 (2011).

7. Gu, Y., Li, D.-W. & Brüschweiler, R. Decoding the Mobility and Time Scales of Protein Loops. *J. Chem. Theory Comput.* **11**, 1308–1314 (2015).

8. Gu, Y., Li, D.-W. & Brüschweiler, R. Statistical database analysis of the role of loop dynamics for protein–protein complex formation and allostery. *Bioinformatics* **33**, 1814–1819 (2017).
